# Associations between serum levels of ferroptosis-related molecules and outcomes in stable COPD: an exploratory prospective observational study

**DOI:** 10.1007/s11739-025-04016-z

**Published:** 2025-06-21

**Authors:** Cristina Ghadban, Mayte García-Unzueta, Juan Agüero, Paula Martín-Audera, Bernardo Alio Lavín, Armando Raúl Guerra, Ana Berja, Nieves Aranda, Anastasia Guzun, Ana Isabel Insua, Carlos Antonio Amado

**Affiliations:** 1 Department of Pulmonology, Hospital Universitario Marqués de Valdecilla, Santander, Spain; 2 University of Cantabria, Santander, Spain; 3 IDIVAL (Instituto de Investigación Biomédica de Cantabria), Cantabria, Spain; 4 Department of Biochemistry, Hospital Universitario Marqués de Valdecilla, Santander, Spain; 5 Department of Biochemistry, Hospital Universitario de Burgos, Burgos, Spain

**Keywords:** COPD, Exacerbation, Ferroptosis, Exercise capacity, STfR1, GPX4, AIFM2

## Abstract

Ferroptosis is an iron-dependent form of cell death that contributes to the pathophysiology of chronic obstructive pulmonary disease (COPD). Ferroptosis-associated factors, including acyl-CoA synthetase long-chain family 4 (ACSL4), soluble transferrin receptor 1 (sTfR1), glutathione peroxidase 4 (GPX4), and apoptosis-inducing factor 2, (AIFM2) mediate intracellular iron metabolism, oxidative stress, and lipid peroxidation. Despite their potential clinical relevance, no studies have measured serum levels of these factors with respect to the manifestations of COPD. The study enrolled 179 stable, non-anemic outpatients diagnosed with COPD and 57 age- and sex-matched smokers who did not carry this diagnosis. Clinical characteristics were assessed together with baseline serum levels of the four ferroptosis-associated factors. Moderate and severe exacerbations of COPD were monitored over the following 12 months. Soluble TfR1 levels were higher and GPX4 levels were lower among those in the COPD group compared to smokers without COPD (*p* = 0.004 and *p* = 0.002, respectively). The sTfR1/GPX4 ratio was also higher among those in the COPD group (*p* = 0.001). Multivariate analysis identified low serum GPX4 (OR 5.475; *p* = 0.001), and high sTfR1/GPX4 (OR 4.293; *p* < 0.001) as independent predictors of poor performance on the six-minute walk distance test. Additionally, high sTfR1 (HR 1.850; *p* = 0.004), low GPX4 (HR 2.301; *p* = 0.001), and high sTfR1/GPX4 (HR 2.223; *p* < 0.001) were associated with increased risk of moderate exacerbation. High sTfR1 (HR 2.970; *p* = 0.014), low GPX4 (HR 3.753; *p* = 0.012), and high sTfR1/GPX4 (HR 3.668; *p* = 0.009) were also independent predictors of severe exacerbation. Serum levels of ferroptosis-associated factors were significantly different in patients diagnosed with COPD compared to smokers who had not been diagnosed with this disorder. Elevated sTfR1, low levels of GPX4, and higher sTfR1/GPX4 were associated with poor clinical outcomes, including reduced exercise capacity and an increased risk of moderate and severe exacerbations. These findings highlight the potential of ferroptosis-associated factors, particularly the calculated sTfR1/GPX4, in predicting COPD progression and the risk of exacerbation in stable, non-anemic outpatients.

**Supplementary Information:**

The online version contains supplementary material available at 10.1007/s11739-025-04016-z.

## Introduction

Chronic Obstructive Pulmonary Disease (COPD) is a smoking-related disorder characterized by progressive and poorly reversible airway obstruction that is variably associated with the destruction of the lung parenchyma [1]. COPD is associated with changes in several variables, including diminished exercise capacity [2] and the development of exacerbations [3]. These factors have an impact on the long-term survival of patients with COPD. In addition, non-anemic iron deficiency (NAID), a condition frequently underdiagnosed in COPD, has been linked to impaired exercise capacity, reduced response to pulmonary rehabilitation, and increased pulmonary artery pressure [4–6].

Ferroptosis is a type of iron-dependent programmed cell death [7]. Lung epithelial cells exposed to cigarette smoke accumulate unstable iron and increase lipid peroxidation. These events are frequently accompanied by ferroptosis, a non-apoptotic form of cell death [8]. Results from several in vivo studies have revealed that ferroptosis plays also an important role in the pathogenesis of specific respiratory infections [9–12]. Cigarette smoke and respiratory infections have been associated with the development of COPD.

Ferroptosis is regulated by a variety of factors that can be measured in human serum. Among these, acyl-CoA synthetase long-chain family member 4 (ACSL4) is an enzyme that converts fatty acids to fatty acyl-CoA esters, regulates lipid biosynthesis, and initiates the intracellular synthesis of phospholipids that undergo lipid peroxidation. ACSL4 has been closely related to ferroptosis in several experimental studies [13, 14]; levels of this factor have been evaluated in animal and human cell models of COPD, but not in human serum [15, 16]. Transferrin receptor 1 (TfR1) has been detected in nearly all proliferative cells where it binds monoferric or diferric iron and mediates cellular iron uptake. TfR1 function is an essential initiator of ferroptosis and is considered a specific ferroptosis marker [17]. Circulating or soluble TfR1 (sTfR1) is a cleaved monomeric form [18] that serves as a surrogate marker of the membrane TfR1 levels, as its concentration is proportional to the total body TfR1 content [18, 19]. Interestingly, sTfR1 levels are less prominently affected by inflammation than ferritin. Of note, results from recent studies have revealed that sTfR1 levels can predict all-cause mortality regardless of anemia and iron storage status [19], being also a marker of chronic diseases [19]. Although sTfR1 has been measured in COPD patients as an indicator of functional iron status [20], its levels have not been studied with respect to other ferroptosis-related molecules or COPD exacerbations. The enzyme glutathione peroxidase 4 (GPX4) plays a critical role in blocking ferroptosis by eliminating phospholipid hydroperoxides and is considered the main inhibitor of ferroptosis [21]. GPX4 has been measured in COPD mouse models [7, 22, 23] as well as in lung tissue collected from patients with COPD [24, 25], but not in serum. Low serum GPX4 levels have been identified in other clinical conditions that have been associated with ferroptosis [26, 27]. Apoptosis-inducing factor mitochondria-associated 2 (AIFM2) is a GPX4-independent suppressor of ferroptosis [28, 29]. AIFM2 is negatively regulated by exposure to cigarette smoke in vitro [30] but has not been studied in serum samples from COPD patients. There are no published studies that have simultaneously measured serum levels of all four of these ferroptosis-associated factors in individuals diagnosed with COPD.

We hypothesized that serum levels of ACSL4, sTfR1, AIFM2, and GPX4 would be altered in COPD. Furthermore, we anticipated that these changes would be linked to outcomes related to exercise capacity, thus increasing the risk of disease exacerbations. This study is an exploratory, clinically oriented investigation.

## Methods

This study was a prospective, observational investigation conducted from October 2018 to September 2023 at a COPD outpatient clinic within a tertiary hospital in Spain. Samples and data from patients included in this study were preserved by the Biobank Valdecilla (PT17/0015/0019). COPD collection was approved by the Ethics Committee of our institution (2018.189). The study protocol is registered on ClinicalTrials.gov (https://clinicaltrials.gov/study/NCT06102993) and was approved by the Ethics Committee of our institution (approval number 2023.297). Written informed consent was obtained from all participants prior to enrollment.

### Participants

Individuals diagnosed with COPD were randomly recruited during routine visits to the dedicated outpatient clinic. Age- and sex-matched control subjects, defined as smokers who had not been diagnosed with COPD, were recruited from the smoking cessation clinics at our institution.

The inclusion criteria for the first group were a diagnosis of COPD, as defined by the Global Initiative for Chronic Obstructive Lung Disease (GOLD) Guidelines (i.e., patients with a history of smoking and a post-bronchodilator forced spirometry with a ratio of < 0.7) who were 40 years of age or older. The control group included age- and sex-matched smokers without COPD.

Exclusion criteria included: (1) history of a COPD exacerbation within 8 weeks prior to study inclusion, (2) ongoing pulmonary rehabilitation treatment or rehabilitation within the 6 months preceding inclusion, (3) a history of coronary artery disease or cancer, (4) polycythemia (hemoglobin > 18 g/dL), anemia (hemoglobin < 12 g/dL), or genetic disorders affecting iron metabolism (e.g., hemochromatosis), and (5) individuals with a glomerular filtration rate < 50 mL/min/1.73m^2^.

### Measurements

Spirometry and a six-minute walk distance (6MWD) test were performed following the protocols established by the Spanish Society of Pulmonology and Thoracic Surgery [31, 32]. Body composition was assessed using a bioelectrical impedance device (OMRON BF511, Omron, Japan). Maximum hand grip strength was measured with a GRIP-A hand dynamometer (Takei, Niigata, Japan). At baseline, patients were classified as having a high risk of exacerbation if they had experienced two or more moderate exacerbations or one severe exacerbation, as per GOLD guidelines, during the previous year (1). Current treatment for COPD, including inhaled corticosteroid use, was also recorded. Patients were classified according to GOLD stages (1–4) and groups (A–E) (1). Modified Medical Research Council (mMRC) Dyspnea Scale scores were also assessed. Smoking history (current or former smoker) was documented, and the Charlson Comorbidity Index was calculated. A COPD Assessment Test (CAT) was performed to assess symptom burden. Oxygen desaturation (OD) was defined as a decrease in oxygen saturation (SpO₂) of ≥ 4% or a SpO₂ level < 90% [33]. Serum creatinine, albumin, uric acid, creatine kinase, iron, ferritin, transferrin, and transferrin saturation levels were measured using Siemens traceable enzymatic method assays (Atellica Analyzer, Siemens, Germany).

Levels of ACSL4, sTfR1, AIFM2, and GPX4 were assessed quantitatively using specific sandwich immunoassays, including the Human AIFM2 ELISA Kit (MBS7246204, MyBioSource, CA, USA), Human GPX4 ELISA Kit (EH8916, FineTest, China), Human ACSL4 ELISA Kit (EH6088, FineTest, China), and Human sTfR1 ELISA Kit (EH0386, FineTest, China) as per the manufacturer’s instructions. We tested the potential utility of a serum sTfR1/GPX4 ratio [the calculated ratio of serum sTFR1 (a pro-ferroptosis molecule) to serum GPX4 (an anti-ferroptosis molecule) multiplied by 1000].

Early morning blood samples were collected from each participant after written informed consent to participate in the study was provided. All samples and associated data were stored by the Biobank Valdecilla (PT17/0015/0019), which is part of the Spanish Biobank Network. The samples were processed according to standard operating procedures.

Following enrollment, participants were monitored for 12 months. During this period, moderate COPD exacerbation (defined as exacerbations requiring treatment with antibiotics and/or systemic corticosteroids) and hospitalizations due to severe COPD exacerbation were recorded prospectively. Additional information was gathered through patient reports at follow-up visits (6 and 12 months post-enrollment) and from medical records from hospital and primary care visits. Exacerbations and hospitalization decisions were made by general or emergency physicians who were responsible for clinical diagnosis and management and were not involved in this study. 

### Statistical analysis

Data are presented as mean ± standard deviation (SD) for normally distributed variables or as medians (interquartile ranges) for nonparametric data. The sample size was calculated using Stata Statistical Software: Release 15 (StataCorp LLC, College Station, TX, USA), with an *α* risk of 0.05 and a *β* risk of 0.2. Differences between groups were assessed using unpaired t tests for parametric data and Mann–Whitney tests for nonparametric data. The Kolmogorov–Smirnov test was used to assess the normality of the distribution.

For the evaluation of serum levels of ACSL4, sTfR1, AIFM2, and GPX4 as dichotomized variables, a cut-off at the median provided the best discriminative power for our outcomes, as indicated by the lowest Akaike Information Criterion value, and consistent with findings from similar studies [34, 35]. The 6MWD test cut-off was set at 350 m, based on the body mass index, airflow obstruction, dyspnea, and exercise capacity (BODE) index.

Cross-sectional associations were evaluated using both univariate and multivariate logistic regression with high vs. low ferroptosis-associated factors and 6MWD test scores as the outcome variables. We used Kaplan–Meier estimates to assess the proportion of participants experiencing an event over time.

Univariate and multivariate analyses were conducted using Cox proportional hazards regression with Statistical Package for the Social Sciences Software version 25.0 (IBM, Armonk, NY, USA) to identify risk factors associated with moderate and severe COPD exacerbations. All reported p values are two-sided. A p value of less than 0.05 was considered statistically significant. 

## Results

### Characteristics of patients and controls

We enrolled 179 stable, non-anemic outpatients diagnosed with COPD and 57 sex- and age-matched controls (Supplementary file 1) (Table 1). All included COPD patients were in a stable clinical condition and met predefined exclusion criteria including anemia and recent exacerbation. Table 1 includes demographic, clinical, and biochemical data. The median age of the patients was 67 (61–72) years and 67.6% were men. All participants in the study were current or former smokers. The COPD group included a high prevalence of current smokers [62 patients (34.6%)], this prevalence was also high in the control group [26 controls (45.6%) were current smokers]. No statistical differences in smoking habit were found between both groups (*p* = 0.136). Most of the participants had moderate or severe airway obstruction. Participants in the control group had lower Charlson Comorbidity Indices and CAT scores, no apparent alterations in lung function, and higher scores on the 6MWD test than were observed among participants diagnosed with COPD. No differences were found in the serum levels of iron, ferritin, transferrin, and transferrin saturation between participants in the COPD and control groups.

**Table 1:** Demographic, clinical and biochemical characteristics of controls and COPD patients

Variable	COPD *n* = 179	Control group *n* = 57	*p*
Age (years)	67 (61–72)	65 (62–69)	0.232
Sex male, *n* (%)	121 (67.6)	36 (63.2)	0.536
Current smokers, *n* (%)	62 (34.6)	26 (45.6)	0.136
FVC (mL)	**2968 ± 861**	**3447 ± 895**	** < 0.001**
FVC (%)	**89 (74–102)**	**101 (89–116)**	** < 0.001**
FEV_1_ (mL)	**1471 ± 614**	**2673 ± 676**	** < 0.001**
FEV_1_ (%)	**56.5 ± 20.9**	**101.0 ± 16.7**	** < 0.001**
FEV_1_/FVC	**48 (38–60)**	**75 (72–79)**	** < 0.001**
Weight (Kg)	77.4 ± 43.4	76.4 ± 16.7	0.867
BMI (Kg/m2)	27.4 ± 4.8	27.8 ± 5.1	0.668
6MWD (m)	**413 ± 114**	**509 ± 106**	** < 0.001**
Maximum hand grip strength (Kg)	30.1 ± 7.9	31.9 ± 10.1	0.345
FFMI (Kg/m^2^)	18.7 ± 2.9	19.2 ± 2.6	0.465
CAT score	**9 (4–16)**	**3 (1–5)**	** < 0.001**
Charlson	**1 (1–2)**	**1(0–2)**	**0.036**
mMRC score 0/I/II/III/IV *n* (%)	**63 (35)/52(29)/46(25)/18(10)**	**45 (78)/8 (14)/4 (7)/0 (0)**	** < 0.001**
Current smokers, *n* (%)	62 (34)	26(45)	0.136
GOLD 1/2/3/4, *n* (%)	23(13.4)/84(46.9)/53(29.6)/18(10.1)	-	-
GOLD A/B/E, *n* (%)	42 (23.5)/55(30.7)/82(45.8)	-	-
1 or more admissions in the previous year, *n* (%)	56 (31.3)	-	-
ICS treatment, *n* (%)	67 (37.4)	-	
Diabetes mellitus, *n* (%)	21 (11.7)	6 (10.5)	0.803
Cardiovascular disease, *n* (%)	22 (12.3)	2 (3.5)	0.056
Albumin (g/dL)	4.84 ± 3.2	4.71 ± 0.25	0.764
Creatinine (mg/dL)	0.81 ± 0.22	0.82 ± 0.18	0.674
Uric acid (mg/dL)	5.7 (4.5–6.7)	5.2 (4.8–6.3)	0.494
CK (UI/L)	83 (56–127)	72 (48–115)	0.141
Hemoglobin (g/dL)	14.6 ± 1.2	14.7 ± 2.1	0.637
Iron (µg/dL)	95 (75–119)	94 (79–114)	0.910
Ferritin (ng/mL)	86 (40–183)	95.8 (39.4–198.4)	0.631
Transferrin (mg/dL)	251 (226–274)	242 (221–272)	0.229
Transferrin saturation (%)	26.4 (20.7–34.4)	26.9 (22.6–33.9)	0.508
ACSL4 (pg/mL)	99.6 (32.35–305.7)	92.8 (48.2–391.3)	0.553
sTfR1 (mg/L)	**2.231 (1.395–3.455)**	**1.813 (0.933–2.796)**	**0.004**
AIFM2 (ng/mL)	7 (5.6–9)	7.4 (5.4–9.2)	0.725
GPX4 (pg/mL)	**78.1 (78.1–213.8)**	**12,307.4 (231.8–23,725.99)**	**0.002**
sTfR1/GPX4	**19.82 (9.91–38.52)**	**12.31 (0.23–23.73)**	**0.001**

*ACSL*4 acyl-CoA synthetase long-chain family member 4, *sTfR*1 soluble transferrin receptor-1, *AIFM*2 apoptosis-inducing factor mitochondria-associated 2, *GPX*4 glutathione peroxidase 4, *FVC* forced vital capacity, *FEV*1 forced expiratory volume in the first second, *mMRC* modified medical research council dyspnea score, *CAT* COPD assessment test, *sTfR*1/*GPX*4 ratio (sTRF1 X 10^3^)/GPX4, *ICS* Inhaled Corticosteroids, *GOLD* global initiative for chronic obstructive lung disease, *BMI* body mass index, *FFMI* fat free mass index, 6*MWD* 6 min walk test distance, *CRP* C-reactive protein, Bold font indicates statistical significance

### Baseline serum levels of ferroptosis-associated factors

The median serum level of sTfR1 level was higher in the COPD group, at 2.231 (1.395–3.455) mg/L compared to the control group, at 1.813 (0.933–2.796) mg/L (*p* = 0.004). Serum GPX4 levels measured in the control group varied substantially; by contrast, the range was markedly smaller among those in the COPD group, with many of the measurements falling below the level of sensitivity of the assay (78.1 pg/mL). Nevertheless, we concluded that the median serum GPX4 level was lower in the COPD group, at 78.1 (78.1–213.8) pg/mL compared to the control group at 12,307.4 (231.8–23,725.99) pg/mL (*p* = 0.002). The median calculated sTfR1/GPX4 was higher in the COPD group, at 19.82 (9.91–38.52) compared to the control group, at 12.31 (0.23–23.73) (*p* = 0.001). We found no inter-group differences in the median serum levels of ACSL4 (*p* = 0.553) or AIFM2 (*p* = 0.725).

We evaluated the correlations of the different ferroptosis-associated factors in COPD. We identified a positive correlation between serum levels of ACSL4 and GPX4 (*p* < 0.001 r = 0.330), and a negative correlation between serum levels of ACSL4 and AIFM2 (*p* < 0.001 *r* = − 0.320). We also identified a negative correlation between serum levels of sTfR1 and GPX4 (*p* < 0.001, *r* = − 0.264). We found no other correlations between serum levels of ferroptosis factors. As expected, we found significant (albeit weak) correlations between serum levels of sTfR1 and parameters associated with iron metabolism, including transferrin (*p* = 0.019, *r* = 0.176), iron (*p* = 0.006, *r* = − 0.204), ferritin (*p* = 0.023, *r* = − 0.171), and transferrin saturation (*p* = 0.001, *r* = − 0.240), and no significant correlation was found between sTFR1 and hemoglobin (*p* = 0.691, *r* = 0.029). We identified no correlations between serum levels of GPX4 and factors involved in iron metabolism. Notably, none of the ferroptosis-related molecules were associated with smoking status.

### Baseline associations of serum levels of ferroptosis-associated factors with characteristics of COPD

Associations of ferroptosis-associated factors with baseline COPD characteristics are shown in Table 2. The results of univariate logistic regression revealed that high serum levels of ACSL4 were associated with female sex, while high serum levels of AIFM2 were associated with the mMRC dyspnea score and negatively associated with forced vital capacity and transferrin saturation. sTFR1 levels were negatively associated with transferrin saturation. High serum levels of GPX4 were positively associated with fat-free mass index (FFMI) and negatively associated with previous exacerbations. High values of sTfR1/GPX4 were positively associated with previous exacerbations and negatively associated with 6MWD test scores and FFMI.

**Table 2:** Unadjusted and adjusted associations between chronic obstructive pulmonary disease characteristics and high levels of ferroptosis molecules (≥ median) using uni- and multivariate logistic regression. (**a**) Model A: Univariate logistic regression. (**b**) Model B: Multivariate logistic regression

		ACSL4		sTfR1		AIFM2		GPX4		sTfR1/GPX4	
		OR (95% CI)	*p*	OR (95% CI)	*p*	OR (95% CI)	*p*	OR (95% CI)	*p*	OR (95% CI)	*p*
(a) Model A: univariate logistic regression											
Age (years)		1.011 (0.974–1.049)	0.573	1.014 (0.976–1.053)	0.466	0.990 (0.953–1.028)	0.590	1.019 (0.980–1.060)	0.336	1.013 (0.976–1.052)	0.499
Sex											
	Male	1		1		1		1		1	
	Female	**1.981 (1.040–3.773)**	**0.038**	1.089 (0.582–2.038)	0.789	0.588 (0.312–1.108)	0.588	1.119 (0.585–2.140)	0.734	1.042)0.555–1.957)	0.897
Smoking status											
	Former	1		1		1		1		1	
	Current	0.904 (0.484–1.686)	0.750	0.891 (0.481–1.649)	0.712	1.085 (0.586–2.009)	0.795	1.311 (0.689–2.493)	0.409	0.964 (0.518–1.793)	0.909
Exacerbation											
	0–1	1		1		1		1		1	
	> 1	1.207 (0.661–2.203)	0.540	0.646 (0.354–1.177)	0.153	1.028 (0.566–1.866)	0.928	**0.506 (0.268–0.956)**	**0.036**	**2.695 (1.437–5.050)**	**0.002**
mMRC Dyspnea score		1.119 (0.834–1.502)	0.454	1.185 (0.882–1.591)	0.260	**1.460 (1.078–1.977)**	**0.014**	1.261 (0.931–1.706)	0.134	0.901 (0.671–1.210)	0.488
Charlson		0.908 (0.639–1.291)	0.592	0.894 (0.598–1.205)	0.360	1.127 (0.796–1.597)	0.500	0.875 (0.608–1.261)	0.475	0,965 (0.681–1.367)	0.841
FFMI (kg/m2)		1.022 (0.924–1.130)	0.673	0.904 (0.816–1.002)	0.056	0.959 (0.851–1.082)	0.496	**1.113 (1.001–1.238)**	**0.047**	**0.838 (0.752–0.934)**	**0.001**
6MWD (m)		0.999 (0.996–1.001)	0.333	**0.997 (0.995–1.000)**	**0.022**	1.000 (0.998–1.002)	0.931	1.001 (0.999–1.004)	0.273	**0.997 (0.994–1.000)**	**0.020**
FEV1 (%)		1.000 (0.986–1.014)	0.991	0.994 (0.980–1.008)	0.377	0.988 (0.974–1.002)	0.092	0.991 (0.976–1.005)	0.211	1.000 (0.986–1.015)	0.970
FVC (%)		1.011 (0.996–1.027)	0.156	0.998 (0.983–1.013)	0.785	**0.982 (0.967–0.997)**	**0.022**	0.991 (0.975–1.006)	0.245	1.010 (0.995–1.026)	0.195
Ferritin (ng/mL)		1.001 (0.999–1.003)	0.351	0.998 (0.996–1.000)	0.069	1.000 (0.998–1.001)	0.667	1.002 (1.000–1.003)	0.099	0.998 (0.996–1.000)	0.060
Transferrin saturation (%)		1.014 (0.991–1.037)	0.240	**0.954 (0.930–0.978)**	** < 0.001**	**0.954 (0.930–0.979)**	** < 0.001**	1.016 (0.993–1.040)	0.172	0.977 (0.955–1.000)	0.055
(b) Model B: multivariate logistic regression											
Age (years)		1.006 (0.962–1.051)	0.808	0.998(0.954–1.045)	0.939	0.994 (0.950–1.041)	0.803	1.040 (0.991–1.091)	0.111	0.993 (0.947–1.042)	0.777
Sex											
	Male	1		1		1		1		1	
	Female	1.701 (0.795–3.640)	0.171	1.831 (0.845–3.965)	0.125	0.718 (0.334–1.547)	0.398	0.584 (0.260–1.325)	0.200	2.085 (0.917–4.740)	0.079
Smoking status											
	Former	1		1		1		1		1	
	Current	0.751 (0.368–1.535	0.433	0.667 (0.333–1.382)	0.276	0.849 (0.408–1.764)	0.660	0.952 (0.444–2.040)	0.899	0.999 (0.466–2.143)	0.998
Exacerbation											
	0–1	1		1		1		1		1	
	> 1	0.776 (0.397–1.514)	0.457	1.378 (0.692–2.743)	0.361	0.830 (0.416–1.654)	0.596	**0.480 (0.232 −0.994)**	**0.048**	**2.709 (1.308–5.609)**	**0.007**
mMRC dyspnea score		1.095 (0.740–1.620)	0.651	0.906 (0.611–1.342)	0.622	**1.508 (1.007–2.257)**	**0.046**	1.435 (0.952–2.162)	0.084	0.668 (0.442–1.012)	0.057
Charlson		0.940 (0.637–1.388)	0.756	0.771 (0.515–1.154)	0.206	1.089 (0.732–1.621)	0.672	0.849 (0.563–1.278)	0.432	1.075 (0.713–1.621)	0.729
FFMI (kg/m2)		0.994 (0.880–1.122)	0.921	0.941 (0.830–1.067)	0.343	0.989 (0.872–1.123)	0.869	**1.151 (1.007–1.316)**	**0.040**	**0.812 (0.706–0.934)**	**0.004**
6MWD (m)		0.998 (0.995 −1.001)	0.173	0.997 (994–1.000)	0.080	1.003 (0.999–1.006)	0.103	1.003 (1.000–1.006)	0.084	**0.998 (0.971–1.000)**	**0.045**
FEV1 (%)		0.989 (0.964–1.049)	0.368	0.999 (0.974–1.025)	0.953	0.999 (0.973–1.026)	0.959	0.983 (0.957–1.010)	0.214	0.998 q(0.971–1.025)	0.866
FVC (%)		1.026 (0.999–1.053)	0.055	1.005 (0.980–1.031)	0.690	0.987 (0.962–1.013)	0.327	1.000 (0.975–1.026)	0.980	1.020 (0.993–1.048)	0.152
Ferritin (ng/mL)		1.003 (1.000–1.005)	0.054	1.000 (0.998–1.003)	0.885	0.999 (0.997–1.002)	0.663	1.002 (0.999–1.005)	0.121	0.999 (0.996–1.002)	0.462
Transferrin saturation (%)		0.994 (0.964–1.026)	0.719	**0.937 (0.904–0.971)**	** < 0.001**	**0.958 (0.926–0.991)**	**0.014**	1.002 (0.970–1.036)	0.882	0.979 (0.947–1.012)	0.208

*ACSL*4 acyl-CoA synthetase long-chain family member 4, *sTfR*1 soluble transferrin receptor-1, *AIFM*2 apoptosis-inducing factor mitochondria-associated 2, *GPX*4 glutathione peroxidase 4, *sTfR*1/*GPX*4 ratio (sTRF1 X 10^3^)/GPX4, *Exacerbations* need for antibiotic or systemic corticosteroids, 6*MWD* 6 min walk test distance, *FEV*1 forced expiratory volume in the first second, *FVC* forced vital capacity, *FFMI* fat free mass index, Bold font indicates statistical significance

The results of multivariable logistic regression (Table 2) revealed that high serum levels of sTFR1 were negatively associated with transferrin saturation (OR 0.937; 95% Confidence Interval [CI] 0.904–0.971; *p* < 0.001). High serum levels of AIFM2 were associated with mMRC dyspnea scores (OR 1.508; 95% CI 1.007–2.257; *p* = 0.046) and transferrin saturation (OR 0.958; 95% CI 0.926–0.991; *p* = 0.014). High serum levels of GPX4 were associated with FFMI (OR 1.151; 95% CI 1.007–1.316; *p* = 0.040) and previous exacerbations (OR 0.480; 95% CI 0.232–0.994; *p* = 0.048). High sTfR1/GPX4 was also associated with previous exacerbations (OR 2.709; 95% CI 1.308–5.609; *p* = 0.007), FFMI (OR 0.812; 95% CI 0.706–0.934; *p* = 0.004), and 6MWD test scores (OR 0.998; 95% CI 0.971–1.000; *p* = 0.045).

### Baseline serum levels of ferroptosis-associated factors as predictors of 6MWD test scores

Forty-six participants in the COPD group walked less than 350 m in the 6MWD test, including 35 individuals with low serum GPX4 levels, 30 with high serum sTfR1 levels, 25 with high serum AIFM2 levels, 25 with high serum ACSL4 levels, and 35 with high sTfR1/GPX4. Univariate logistic regression revealed that low serum GPX4 levels (*p* = 0.025), high serum sTfR1 levels (*p* = 0.016), and high sTfR1/GPX4 (*p* = 0.002) were predictors of low 6MWD test scores. Absolute values of serum sTfR1 levels (*p* < 0.001) and sTfR1/GPX4 (*p* < 0.001) were also predictors of low 6MWD test scores. By contrast, absolute and dichotomized serum levels of ACSL4 and AIFM2 were not identified as predictors of low 6MWD test scores.

The results of multivariate logistic regression analysis (Table 3) revealed that low levels of serum GPX4 (OR 5.475; 95% CI 2.000–14.991; *p* = 0.001), and high sTfR1/GPX4 (OR 4.293; 95% CI 1.728–10.665; *p* < 0.001), as well as absolute levels of sTfR1 (OR 1.000; (95% CI 1.000–1.000; *p* < 0.001) and sTfR1/GPX4 (OR 1.00; 95% CI 1.000–1.000; *p < *0.001) were predictors of the capacity to walk less than 350 m in the 6MWD test. Using the same model, neither absolute nor dichotomized values of serum levels of ACSL4 or AIFM2 were independent predictors of low 6MWD test scores. Dichotomized values of sTFR1 (OR 2.159; 95% CI 0.927–5.024; *p* = 0.074) suggested a tendency toward walking less than 350 m in the 6MWD test.

**Table 3:** Ferroptosis molecules as predictors in 6-min walk test (6MWT)

	Predictors of walking less than 350 m in 6MWT				
	B	*p*	HR	95% IC HR	
				Inferior	Superior
ACSL4 (pg/mL)	−0.001	0.110	0.999	0.998	1.000
sTfR1 (mg/L)	**0.000**	** < 0.001**	**1.000**	**1.000**	**1.001**
AIFM2 (ng/mL)	0.024	0.697	1.024	0.947	1.030
GPX4 (pg/mL)	0.000	0.203	1.000	0.999	1.000
sTfR1/GPX4	**0.000**	** < 0.001**	**1.00**	**1.000**	**1.001**
High ACSL4	0.166	0.590	1.180	0.527	2.642
High sTfR1	0.769	0.074	2.159	0.927	5.024
High AIFM2	0.062	0.885	1.064	0.459	1.031
Low GPX4	**1.700**	**0.001**	**5.475**	**2.000**	**14.991**
High sTfR1/GPX4	**1.457**	**0.002**	**4.293**	**1.728**	**10.665**

	Predictors of oxygen desaturation in 6MWT				
	B	*p*	HR	95% IC HR	
				Inferior	Superior
ACSL4 (pg/mL)	− 0.001	0.314	0.999	0.998	1.001
sTfR1 (mg/L)	**0.000**	**0.014**	**1.000**	**1.000**	**1.000**
AIFM2 (ng/mL)	0.103	0.091	1.109	0.984	1.010
GPX4 (pg/mL)	0.000	0.384	1.000	1.000	1.000
sTfR1/GPX4	**0.000**	**0.001**	**1.000**	**1.000**	**1.000**
High ACSL4	− 0.249	0.549	0.780	0.346	1.760
High sTfR1	**1.378**	**0.003**	**3.974**	**1.583**	**9.978**
High AIFM2	− 0.637	0.146	0.529	0.224	1.248
Low GPX4	− 0.566	0.207	0.568	0.236	1.369
High sTfR1/GPX4	**2.257**	**0.009**	**3.324**	**1.349**	**8.190**

All variables adjusted by Age, Sex, Charlson Index, High risk of exacerbation (2 or more exacerbations during previous year or 1 previous admission), FEV1, Forced Expiratory Volume in the first second, smoking status, ferritin and transferrin saturation. 6*MWD* 6-min walking distance. *ACSL*4 acyl-CoA synthetase long-chain family member 4, *sTfR*1 soluble transferrin receptor-1, *AIFM*2 apoptosis-inducing factor mitochondria-associated 2, *GPX*4 glutathione peroxidase 4, *sTfR*1/*GPX*4 ratio (sTRF1 X 10^3^)/GPX4. High ACSL4 ≥ median for the COPD group, High sTfR1 ≥ median for the COPD group, High AIFM2 ≥ median for the COPD group, High GPX4 ≥ median for the COPD group, High sTfR1/GPX4 ≥ median for the COPD group. Oxygen desaturation (OD) was defined as ≥ 4% reduction between pretest and posttest arterial oxygen saturation (Δ SpO2 ≥ 4%) and posttest SpO2 < 90% measured by pulse oximetry. Bold font indicates statistical significance

Forty-seven participants presented with OD, including 30 with low serum levels of GPX4, 32 with high serum levels of sTfR1, 24 with high serum levels of AIFM2, 21 with high serum levels of ACSL4, and 32 with high sTfR1/GPX4. The results of univariate logistic regression revealed that high serum levels of sTfR1 (*p* = 0.004) and high sTfR1/GPX4 (*p* = 0.048) were predictors of OD. Absolute values of serum AIFM2 (*p* = 0.016) and sTfR1 (*p* = 0.031) levels, as well as sTfR1/GPX4 (*p* = 0.016) were also predictors of OD. By contrast, absolute and dichotomized serum levels of GPX4 and ACSL4 and dichotomized values of serum AIFM2 levels were not predictors of OD.

The results of multivariate logistic regression analysis (Table 3) revealed that high serum levels of sTfR1 (OR 3.974; 95% CI 1.583–9.978; *p* = 0.003) and high sTfR1/GPX4 (OR 3.324; 95% CI 1.349–8.190; *p* = 0.009), as well as absolute values of serum sTfR1 (OR 1.000; 95% CI 1.000–1.000; p = 0.014), and calculated sTfR1/GPX4 (OR 1.00; 95% CI 1.000–1.000; *p* = 0.001) were positively associated with OD. Using the same model, neither absolute nor dichotomized values of serum AIFM2, ACSL4, or GPX4 levels proved to be independent predictors of low 6MWD test scores.

### Baseline serum levels of ferroptosis-associated factors as predictors of moderate exacerbation

During the 12-month follow-up period, 88 of the 179 study participants developed moderate COPD exacerbations, including 72 with low serum levels of GPX4, 57 with high serum levels of sTfR1, 55 with high serum levels of AIFM2, 51 with high serum levels of ACSL4, and 68 with high sTfR1/GPX4.

The results of univariate Cox proportional risk analysis revealed that low serum levels of GPX4 (*p* = 0.017), high levels of sTfR1 (*p* = 0.017), and high sTfR1/GPX4 (p = 0.001), as well as the absolute values of serum sTfR1 (*p* = 0.004) and calculated sTfR1/GPX4 (*p* = 0.009) were potential risk factors for COPD exacerbation. By contrast, high serum levels of AIFM2 or ACSL4 and absolute values of serum GPX4, AIFM2, or ACSL4 levels were not identified as potential risk factors.

**Fig. 1:**
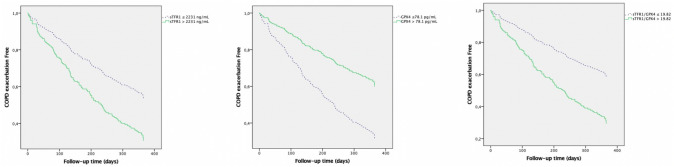
Serum sTfR1, GPX4, and sTFR1/GPX4 levels as predictors of moderate COPD exacerbations

The results of multivariate Cox proportional risk analysis identified low serum levels of GPX4 (HR 2.301; 95% CI; 1.426–3.713; *p* = 0.001), high serum levels of sTfR1 (HR 1.850; 95% CI 1.212–2.823; *p* = 0.004), and high sTfR1/GPX4 (HR 2.223; 95% CI 1.441–3.428; *p* < 0.001), as well as absolute serum levels of GPX4 (HR 1.000; 95% CI 1.000–1.000; *p* = 0.049) and sTfR1 (HR 1.000; 95% CI 1.000–1.001; *p* = 0.001), and sTfR1/GPX4 (HR 1.00; 95% CI 1.000–1.000; *p* = 0.001) as potential independent risk factors for moderate COPD exacerbation (Fig. 1; Table 4). Using the same model, absolute and dichotomized serum levels of ACSL4 and AIFM2 were not identified as potential independent risk factors for moderate COPD exacerbation.

**Table 4:** Ferroptosis molecules as predictors of COPD exacerbations

	Predictors of moderate exacerbation				
	B	*p*	HR	95% IC HR	
				Inferior	Superior
ACSL4 (pg/mL)	0.000	0.873	1.000	0.999	1.001
sTfR1 (mg/L)	**0.000**	**0.001**	**1.000**	**1.000**	**1.001**
AIFM2 (ng/mL)	0.007	0.823	1.007	0.946	1.072
GPX4 (pg/mL)	**0.000**	**0.049**	**1.000**	**1.000**	**1.000**
sTfR1/GPX4	**0.000**	**0.001**	**1.000**	**1.000**	**1.000**
High ACSL4	0.002	0.992	1.002	0.665	1.001
High sTfR1	**0.615**	**0.004**	**1.850**	**1.212**	**2.823**
High AIFM2	0.234	0.291	1.263	0.818	1.949
Low GPX4	**0.833**	**0.001**	**2.301**	**1.426**	**3.713**
High sTfR1/GPX4	**0.799**	** < 0.001**	**2.223**	**1.441**	**3.428**

	Predictors of COPD hospitalization				
	B	*p*	HR	95% IC HR	
				Inferior	Superior
ACSL4 (pg/mL)	0.001	0.587	1.000	0.998	1.001
sTfR1 (mg/L)	**0.000**	**0.049**	**1.000**	**1.000**	**1.000**
AIFM2 (ng/mL)	0.031	0.566	1.031	0.928	1.146
GPX4 (pg/mL)	− 0.001	0.260	0.999	0.997	1.001
sTfR1/GPX4	**0.000**	**0.024**	**1.000**	**1.000**	**1.000**
High ACSL4	− 0.355	0.390	0.701	0.312	1.575
High sTfR1	**1.088**	**0.014**	**2.970**	**1.247**	**7.071**
High AIFM2	0.515	0.224	1.674	0.724	3.869
Low GPX4	**1.323**	**0.012**	**3.753**	**1.339**	**10.521**
High sTfR1/GPX4	**1.300**	**0.009**	**3.668**	**1.380**	**9.750**

All variables adjusted by Age, Sex, Charlson Index, High risk of exacerbation (2 or more exacerbations during previous year or 1 previous admission), FEV1, Forced Expiratory Volume in the first second, smoking status, ferritin, and transferrin saturation. 6*MWD* 6-min walking distance, *ACSL*4 acyl-CoA synthetase long-chain family member 4, *sTfR*1 soluble transferrin receptor-1, *AIFM*2 apoptosis-inducing factor mitochondria-associated 2, *GPX*4 glutathione peroxidase 4, *sTfR*1/*GPX*4 ratio (sTRF1 X 10^3^)/GPX4. High ACSL4 ≥ median for the COPD group, High sTfR1 ≥ median for the COPD group, High AIFM2 ≥ median for the COPD group, High GPX4 ≥ median for the COPD group, High sTfR1/GPX4 ≥ median for the COPD group. Bold font indicates statistical significance

### Baseline serum levels of ferroptosis-associated factors as predictors of severe COPD exacerbation

During the 12-month follow-up period, 30 study participants were hospitalized, including 25 with low serum levels of GPX4, 22 with high serum levels of sTfR1, 20 with high serum levels of AIFM2, 11 with high serum levels of ACSL4, and 25 with high sTfR1/GPX4.

The results of univariate Cox proportional risk analysis revealed that low serum levels of GPX4 (*p* = 0.014), high serum levels of sTfR1 (*p* = 0.005), and high sTfR1/GPX4 (*p* = 0.003), as well as absolute values of serum sTfR1 (*p* < 0.001) and sTfR1/GPX4 (*p* < 0.001) were potential risk factors for COPD-related hospitalization. By contrast, high serum levels of AIFM2 or ACSL4 and absolute values of serum GPX4, AIFM2, or ACSL4 levels were not potential predictors of COPD-related hospitalization.

**Fig. 2:**
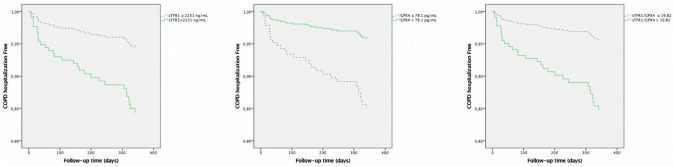
Serum sTfR1, GPX4, and sTFR1/GPX4 levels as predictors of hospitalizations

The results of multivariate Cox proportional risk analysis revealed that low serum levels of GPX4 (HR 3.753; 95% CI 1.339–10.521; *p* = 0.012), high serum levels of sTfR1 (HR 2.970; 95% CI 1.247–7.071; *p* = 0.014), and high sTfR1/GPX4 (HR 3.668; 95% CI 1.380–9.750; *p* = 0.009), as well as absolute serum levels of sTfR1 (HR 1.000; 95% CI 1.000–1.000; *p* = 0.049) and sTfR1/GPX4 (HR 1.00; 95%IC 1.000–1.000; *p* = 0.024) were potential risk factors for COPD-related hospitalization (Fig. 2; Table 4). Using the same model, absolute and dichotomized serum levels of ACSL4 and AIFM2 and absolute serum levels of GPX4 were not identified as potential independent risk factors for COPD-related hospitalization.

## Discussion

Our study is the first to report the results of a simultaneous evaluation of serum levels of four ferroptosis-associated factors in individuals diagnosed with COPD. Our results include several important findings. First, the study participants with stable COPD exhibited distinct alterations in serum levels of ferroptosis-associated factors when compared to controls, specifically, higher levels of sTfR1, lower levels of GPX4, and increased sTfR1/GPX4, while serum levels of ACSL4 and AIFM2 remained unchanged. Additionally, the observed changes in serum levels of ferroptosis-associated factors were associated with key clinical outcomes, including previous exacerbations, exercise capacity as assessed by 6MWD, and the risk of future exacerbation. Furthermore, sTfR1/GPX4 emerged as a potentially valuable biomarker for identifying COPD patients who might be at greater risk of poor outcomes. Finally, our findings suggest that ferroptosis might be an important mechanistic pathway contributing to the pathophysiology of COPD.

The changes observed in serum sTfR1 and GPX4 levels in stable COPD patients compared to non-COPD controls are consistent with the contributions of these two factors to mechanisms underlying ferroptosis, although need further studies. For example, sTfR1 promotes iron entry into the cell, thereby initiating the ferroptosis pathway [7, 17]. The discovery that TfR1 accumulates on the surface of ferroptotic cells, facilitating the extracellular release of sTfR1, is significant in this regard [17]. Recruitment of TfR1 to the plasma membrane is most likely the result of a positive feedback cycle linking iron uptake and ferroptotic cell death, although the precise mechanism remains unknown [17]. By contrast, GPX4 inhibits ferroptosis by eliminating intracellular phospholipid hydroperoxides [7]. Active ferroptosis leads to a decrease in GPX4 levels due to its hyper-consumption. Our findings of high circulating levels of sTfR1 together with low levels of GPX4 are consistent with the hypothesis that ferroptosis may be over-activated in COPD, similar to what has been reported for ferroptosis associated with diabetic nephropathy [26, 27]. It is also interesting to note the significantly negative (albeit somewhat weak) correlation between these factors, findings that suggest that they are in some way connected.

The metabolic processes linking intra- and extracellular iron metabolism in COPD are extremely complex [36] Smoking-induced iron overload in lung tissue is a well-recognized finding associated with COPD. As part of this pathological state, iron content and levels of iron-binding factors are increased in lung tissue, sputum, bronchoalveolar lavage fluid (BAL), and alveolar macrophages [37]. There is also clear evidence of excessive ferroptosis in bronchial epithelial cells, lung macrophages, and pulmonary endothelial cells [38]. Moreover, the results of several recent studies revealed that anti-ferroptotic agents can mitigate the progression of COPD in various experimental models [39–41].

However, Perez-Peiró et al. [19] identified a different subgroup of COPD patients who presented with systemic and muscular iron depletion [19] associated with increased serum sTfR1 levels and low exercise tolerance. Similar observations have been reported by other groups. For instance, non-anemic iron deficiency has been shown to impair the response to pulmonary rehabilitation [4], is prevalent in stable COPD outpatients [5], and may be associated with increased pulmonary artery pressures [6], further supporting the clinical relevance of altered iron homeostasis in COPD. We found that serum sTfR1 levels were negatively associated with performance on the 6MWD test and trended toward a negative association with FFMI. These findings might suggest an association between ferroptosis and low muscle mass and function, as was proposed previously [42]. In patients with high serum levels of sTfR1, this finding also showed an association with OD during exercise, indicating a possible ventilation/perfusion mismatch. Finally, our findings revealed that elevated serum sTfR1 levels may serve as a prognostic marker for moderate and severe exacerbations within the following year.

Although comparatively low cellular GPX4 levels have been reported both in vivo and in in vitro models of COPD [8, 22–25], this study is the first to report low GPX4 levels in serum samples from COPD patients. We also found that higher serum levels of GPX4 were associated with higher FFMIs, and that patients with lower serum levels of GPX4 tend to walk less in the 6MWD test and have a higher risk of developing COPD exacerbations during the following 12 months.

We also evaluated the potential role of the sTfR1/GPX4 ratio as a biomarker of COPD. This ratio includes information about serum sTfR1 levels, which may be indicative of increased intracellular iron demand, coupled with serum GPX4 levels, which may reflect an environment prone to lipid peroxidation and ferroptosis. The relationship between sTfR1/GPX4 and clinical outcomes underscores the potential role of ferroptosis in COPD. Furthermore, the strong links between sTfR1/GPX4 and exercise capacity or exacerbation risk provide a rationale for its further exploration as a biomarker in clinical practice.

We were neither able to find differences in levels of serum ACSL4 or AIFM2 between patients and controls, nor were we able to identify significant associations between clinical disease and these two molecules previously linked to ferroptosis in preclinical models of COPD. This discrepancy might be due to the methodological limitations of these recently developed and minimally evaluated ELISAs, including variability and detection sensitivity. On the other hand, significant changes in ferroptotic cells may not be sufficient to be detected in systemic blood. Future research combining systemic and cellular levels of ferroptotic markers might aim to elucidate the relevance of these and other new ferroptosis markers in the pathogenesis and evolution of COPD. Some of our participants exhibited alterations in extracellular iron metabolism, which enabled us to explore the relationship between ferroptosis-related proteins and systemic iron homeostasis. Notably, both AIFM2 and sTfR1 were independently and negatively associated with transferrin saturation in multivariate analysis, supporting a mechanistic link between ferroptosis and systemic iron regulation in COPD.

Our study has several limitations. Because this was a single-center study, our findings will need to be validated in larger, multicenter studies that include participants with diverse sociodemographic characteristics and comorbidities known to influence ferroptosis. Additional research will be needed to confirm or refute the potential roles of sTfR1, GPX4, sTfR1/GPX4, as well as to monitor how the serum levels of these ferroptosis-associated factors change over time. While we applied stringent criteria to exclude patients with altered pulmonary function or conditions other than COPD that might elevate ferroptosis (e.g., active disease exacerbation, sepsis, severe inflammation, renal insufficiency, alcoholism, and iron-related disorders), our results cannot be entirely generalized to all COPD patients. Additionally, we did not exclude COPD patients with low-grade inflammation and other asymptomatic disorders (e.g., coronary disease, atherosclerosis, and hypertension), although we believe that the impact of these conditions on our results was small. Finally, our study highlights associations but does not establish causality. However, despite these limitations, our findings provide valuable new insights into COPD pathophysiology.

The main strength of our study lies in its prospective design, which was tailored to explore the potential utility of measuring ferroptosis-associated molecules in a group of thoroughly evaluated COPD. These samples are comparatively easy to obtain in contrast to those used in the previous studies of ferroptosis that focused on epithelial tissue and biopsy specimens.

In conclusion, in this exploratory study, we simultaneously evaluated serum levels of four ferroptosis-associated factors (ACSL4, sTfR1, AIFM2, and GPX4) and sTfR1/GPX4 in patients diagnosed with COPD. We found that serum levels of sTfR1 were higher and those of GPX4 were lower among study participants with COPD than was observed in smokers without COPD. Serum levels of these molecules were associated with important clinical outcomes. Among the various ferroptosis-associated factors, serum levels of sTfR1 and GPX4 as well as sTfR1/GPX4 revealed a strong prognostic value and may serve as future risk-associated biomarkers for patients diagnosed with COPD. Additional studies will be needed to confirm these original findings. 

## Supplementary Information

Below is the link to the electronic supplementary material.

Supplementary file1 (JPG 40 KB)

## Data Availability

Requests to access the datasets should be directed to corresponding author.
