# Supplementary figures and images for: Associations between serum levels of ferroptosis-related molecules and outcomes in stable COPD: an exploratory prospective observational study
Source: Intern Emerg Med. 2025 Jun 21;20(6):1761–73. doi: 10.1007/s11739-025-04016-z (PMC12476405; doi:10.1007/s11739-025-04016-z)

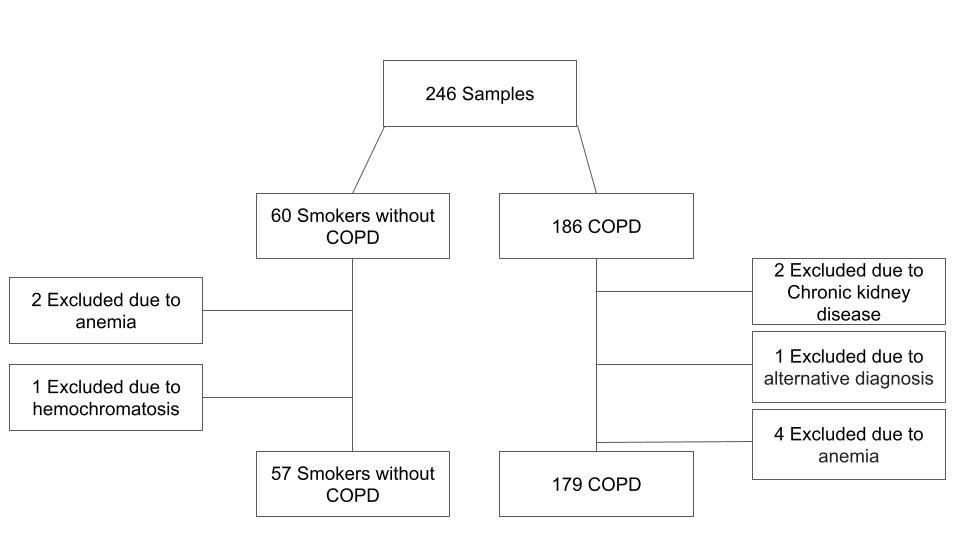

Supplement: Supplementary file 1 — Supplementary file1 (JPG 40 KB) [file 11739_2025_4016_MOESM1_ESM.jpg]
